# Hsa_circ_0004674 promotes osteosarcoma doxorubicin resistance by regulating the miR-342-3p/FBN1 axis

**DOI:** 10.1186/s13018-021-02631-y

**Published:** 2021-08-18

**Authors:** Yumei Bai, Yanghua Li, Juan Bai, Yumei Zhang

**Affiliations:** Department of Pharmacy, Jingmen No.1 People’s Hospital, No.3, Shenzhen Avenue, Duodao District, Jingmen, 448000 Hubei China

**Keywords:** Osteosarcoma, chemoresistance, hsa_circ_0004674, miR-342-3p, FBN1

## Abstract

**Background:**

The occurrence of chemoresistance is a common problem in tumor treatment. Circular RNA (circRNA) has been confirmed to be related to tumor chemoresistance. However, the role and the underlying molecular mechanism of hsa_circ_0004674 in the chemoresistance of osteosarcoma (OS) are still unclear.

**Methods:**

The expression of hsa_circ_0004674, miR-342-3p, and fibrillin-1 (FBN1) was determined by qRT-PCR. Cell counting kit 8 assay was used to evaluate the doxorubicin (DXR) resistance of cells. The proliferation and apoptosis of cells were measured using colony formation assay and flow cytometry. Western blot analysis was utilized to examine the protein levels of resistance markers, Wnt/β-catenin pathway markers and FBN1. The interaction between miR-342-3p and hsa_circ_0004674 or FBN1 was confirmed by dual-luciferase reporter assay and RNA pull-down assay. Moreover, animal experiments were performed to assess the effect of hsa_circ_0004674 silencing on the DXR sensitive of OS in vivo.

**Results:**

The upregulated hsa_circ_0004674 was found in DXR-resistant OS tissues and cells. Knockdown of hsa_circ_0004674 could inhibit the DXR resistance of OS cells in vitro and promote the DXR sensitive of OS tumors in vivo. In addition, we discovered that hsa_circ_0004674 could sponge miR-342-3p, and miR-342-3p could target FBN1. MiR-342-3p inhibitor could reverse the inhibition effect of hsa_circ_0004674 knockdown on the DXR resistance of OS cells. Similarly, the suppressive effect of miR-342-3p on the DXR resistance of OS cells also could be reversed by FBN1 overexpression. Furthermore, we revealed that hsa_circ_0004674 silencing inhibited the activity of Wnt/β-catenin pathway by the miR-342-3p/FBN1 axis.

**Conclusion:**

Hsa_circ_0004674 facilitated the DXR resistance of OS through Wnt/β-catenin pathway via regulating the miR-342-3p/FBN1 axis, suggesting that hsa_circ_0004674 was a promising target for the chemoresistance of OS.

## Introduction

Osteosarcoma (OS) is a malignant connective tissue tumor, and its incidence ranks first among malignant bone tumors [1, 2]. The current treatment of OS is limb salvage under the protection of neoadjuvant chemotherapy, so the sensitivity of chemotherapy has an important impact on the prognosis of OS patients [3, 4]. Drug resistance is a common cause of clinical chemotherapy failure, and its production is a complex biological process, often accompanied by many molecular interactions [5, 6]. Therefore, elucidating the molecular mechanisms affecting chemoresistance is of great significance to the treatment and diagnosis of OS.

Circular RNA (circRNA) contains microRNA (miRNA) binding sites, and can serve as a ceRNA to perform biological functions [7, 8]. Abnormal expression of circRNA can lead to tumorigenesis and changes in cell biological functions [9, 10]. In addition, circRNA has been confirmed to be associated with the chemoresistance of tumors. For example, circ_0026359 could serve as miR-1200 sponge to promote gastric cancer cisplatin resistance [11]. CircHIPK3 was upregulated in glioma, which could enhance the temozolomide resistance of glioma by miR-524-5p/KIF2A axis [12]. Moreover, circKDM4C was discovered to be downregulated in breast cancer, which could reduce the doxorubicin (DXR) resistance of breast cancer [13]. Therefore, circRNA is expected to be a potential target for overcoming the chemoresistance of tumors.

In previous studies, Kun-Peng et al*.* screened out 80 circRNAs that were differentially expressed in chemoresistance and chemosensitive OS cells by using next-generation sequencing analysis [14]. Among them, the significantly high expression of hsa_circ_0004674 has attracted our attention. Nevertheless, the role and molecular mechanism of hsa_circ_0004674 in OS chemoresistance are still unclear. The purpose of our study is to reveal the influence of hsa_circ_0004674 on OS DXR resistance and reveal its underlying molecular mechanism.

## Materials and methods

### Samples

41 primary OS patients were recruited from the Jingmen No.1 People’s Hospital according to the inclusion and exclusion criteria. Inclusion criteria: 1, newly treated patients without previous surgery or chemotherapy; 2, all patients were diagnosed with OS by histopathology; 3, all patients and their family members were informed of this study and signed the consent form. Exclusion criteria: 1, complicated with other tumors; 2, diseases of the nervous system; 3, suffer from serious organ diseases. The correlation between has_circ_0004674 expression and clinicopathological characteristics of OS patients was shown in Table 1. Forty-one tumor tissues from patients with primary OS were obtained, including 18 DXR-resistant OS tissues and 23 DXR-sensitive OS tissues. This study obtained the informed consent from each patient and was approved by Jingmen No.1 People’s Hospital.

**Table 1:** Correlation between has_circ_0004674 expression and clinicopathological characteristics of OS patients (*n* = 41)

Pathological characteristics	Cases (n)	Has_circ_0004674 expression		*P*-value
		High (20)	Low (21)	
Gender				
Male	18	7	11	0.3499
Female	23	13	10	
Age				
≥ 25	16	7	9	0.7513
< 25	25	13	12	
Enneking stage				
I + IIA	13	3	10	0.0431^*^
IIB + III	28	17	11	
Location				
Distal of femur	18	10	8	0.1956
Proximal of tibia	14	8	6	
Other	9	2	7	
Chemoresistant				
Yes	23	8	15	0.0427^*^
No	18	12	6	
Type of disease				
P53 mutation	17	10	7	0.1112
Rb1 mutation	14	8	6	
Other	10	2	8	

^*^P < 0.05

### Cell culture and transfection

Human OS cell lines (U2OS and KHOS) were obtained from Biovector (Beijing China) and were cultured in DMEM (Gibco, Gran Island, NY, USA) containing 10% FBS (Gibco) and 1% penicillin–streptomycin (Invitrogen, Carlsbad, CA, USA) at 37℃ with 5% CO_2_. U2OS and KHOS cells were placed in a medium containing DXR (Zhejiang Hisun Pharmaceutical CO., LTD, Taizhou, China), and the concentration of DXR was gradually increased from 2.5 ng/mL to 1 μg/mL over a period of 6 months as the previously reports [15]. After multiple screenings, DXR-resistant OS cell lines (U2OS_R and KHOS_R) were isolated.

Hsa_circ_0004674 small interference RNA (siRNA) or lentiviral short hairpin RNA (shRNA) (si-hsa_circ_0004674#1/#2/#3 or sh-hsa_circ_0004674), miR-342-3p mimic or inhibitor, pcDNA fibrillin-1 (FBN1) overexpression plasmid (pcDNA-FBN1), and negative controls (si-NC, sh-NC, mimic NC, inhibitor NC and pcDNA) were synthesized from RiboBio (Guangzhou, China). U2OS_R and KHOS_R cells were seeded into 6-well plates (4 × 10^5^ cells per well) and incubated and incubated overnight at 37℃. After the cells reached 50–60% confluences, the above oligonucleotides (40 nM) and vectors (2 μg) were transfected into cells using Lipofectamine 3000 reagent (Invitrogen). Then, the cells were collected for function experiments after transfection for 48 h.

### qRT-PCR

RNA was extracted by TRIzol reagent (Invitrogen) and cDNA was obtained using BeyoRT™ II cDNA Synthesis Kit (Beyotime, Shanghai, China). QRT-PCR was performed by SYBR Green PCR Kit (Qiagen, Duesseldorf, Germany). Relative expression was normalized using β-actin or U6 and expressed using the 2^−ΔΔCT^ method. Primer sequences were shown in Table 2.

**Table 2:** The primer sequences in this study

Gene	Sequences (5’-3’)
hsa_circ_0004674	F: GCTTCCAGGTTGATGCCTTTG
	R: TCCAGGAAGGTTGAAAGAATCCA
ADAM22	F: GAAGACGAAAGTCGGCACGA
	R: TGAATGACGTTCCAAAGGCAT
miR-342-3p	F: GGGTCTCACACAGAAATCGC
	R: CAGTGCGTGTCGTGGAGT
FBN1	F: TTTAGCGTCCTACACGAGCC
	R: CCATCCAGGGCAACAGTAAGC
β-actin	F: CTTCGCGGGCGACGAT
	R: CCACATAGGAATCCTTCTGACC
U6	F: GCTTCGGCAGCACATATACTAAAAT
	R: CGCTTCACGAATTTGCGTGTCAT

### Identification of the circuRNA

Genomic DNA (gDNA) was extracted from U2OS_R and KHOS_R cells using PureLink Genomic DNA Mini Kit (Invitrogen). After amplified with convergent primers and divergent primers using cDNA and gDNA, the PCR products of hsa_circ_0004674, β-actin and ADAM22 were detected by agarose gel electrophoresis. In RNase R assay, the extracted RNAs from U2OS_R and KHOS_R cells were incubated with or without RNase R, and then the RNA was used for measuring hsa_circ_0004674 expression and linear ADAM22 expression.

### Cell counting kit 8 (CCK8) assay

U2OS_R and KHOS_R cells were seeded into 96 well plates (2 × 10^3^ cells/well). Freshly medium containing different concentrations of DXR was added into cells and cultured for 48 h. After that, CCK8 solution (Beyotime) was added into each well and incubated for 4 h. Cell viability was measured at 450 nm to calculate IC_50_.

### Colony formation assay

U2OS_R and KHOS_R cells were seeded in 6-well plates (250 cells/well) and cultured for 14 days. The colonies were stained by crystal violet, and its number was counted under microscope.

### Flow cytometry

After transfection for 48 h, U2OS_R and KHOS_R cells were collected and then centrifuged at 1,000 rpm for 5 min. According to the instructions of Cell Cycle Analysis Kit (Beyotime), the cell suspensions (2 × 10^6^ cells) were fixed with 70% ethanol and stained with RNase A and PI mixtures for 30 min. For detecting cell apoptosis, the cell suspensions (1 × 10^6^ cells) were suspended with binding buffer and stained with Annexin V-FITC (Beyotime) and PI for 15 min. Under flow cytometer, cell cycle process and apoptosis were analyzed using CellQuest software.

### Western blot (WB) analysis

Protein was extracted by RIPA Lysis buffer (Sangon, Shanghai, China), subjected to SDS-PAGE gel, and transferred to PVDF membranes. The membranes were incubated with anti-p-glycoprotein (anti-p-gp, 1:500, Bioss, Beijing, China), anti-myeloid cell leukemia-1 (anti-MCL-1, 1:1,000, Bioss), anti-FBN1 (1:1,000, Bioss), anti-β-catenin (1:2,000, Bioss), anti-c-Myc (1:1,000, Bioss) or anti-β-actin (1:2,000, Bioss). After incubating with Goat Anti-Rabbit IgG (1:20,000, Bioss), the signals were determined by Enhanced ECL Chemiluminescence Detection Kit (Vazyme, Nanjing, China).

### Dual-luciferase reporter assay

The hsa_circ_0004674 or FBN1 3’UTR wt and mut fragments were cloned into the pmirGLO vector. 293 T cells were seeded into 24-well plates at 1 × 10^5^ per well. The vectors (50 ng) were transfected into 293 T cells with miR-342-3p mimic (20 μM) or mimic NC (20 μM). After 48 h, Dual-luciferase Reporter Assay System (Promega, Madison, WI, USA) was applied to analyze the luciferase activity.

### RNA pull-down assay

U2OS_R and KHOS_R cells were transfected with Bio-miR-342-3p probe or Bio-NC probe for 48 h. Then, the cells were lysed and cell lysates were incubated with magnetic beads (Invitrogen), and the bounded RNA was purified using TRIzol reagent. The enrichment of hsa_circ_0004674 and FBN1 was analyzed by qRT-PCR.

### In vivo experiments

Animal experiments were approved by the Animal Ethic Committee of Jingmen No.1 People’s Hospital and performed according to the Guide for the Care and Use of Laboratory Animals. KHOS_R cells were transfected with sh-NC or sh-hsa_circ_0004674 and then subcutaneously injected into the flank of BALB/c nude mice (*n* = 6 per group, 1 × 10^6^ cells/mouse; Vital River, Beijing, China). When the tumor grew about 100 mm^3^, 3 mice in each group were intraperitoneally injected with 2 mg/kg/d DXR, and the remaining mice were injected with the same amount of PBS (Beyotime). The tumor volume was determined by detecting tumor length and width every 4 days. 16 days later, the tumor was removed for weighting and measuring protein expression.

### Statistical analysis

All data were presented as mean ± SD. All experiment was performed in triplicate, and all independent experiments were set for 3 times to take the average value. GraphPad Prism 5.0 software was utilized for the analysis. Student’s *t*-test or ANOVA was used for statistical analysis. Correlations were analyzed by Pearson’s correlation coefficient. *P* < 0.05 was considered as significant.

## Results

### Hsa_circ_0004674 was highly expressed in DXR-resistant OS tissues and cells

**Fig. 1:**
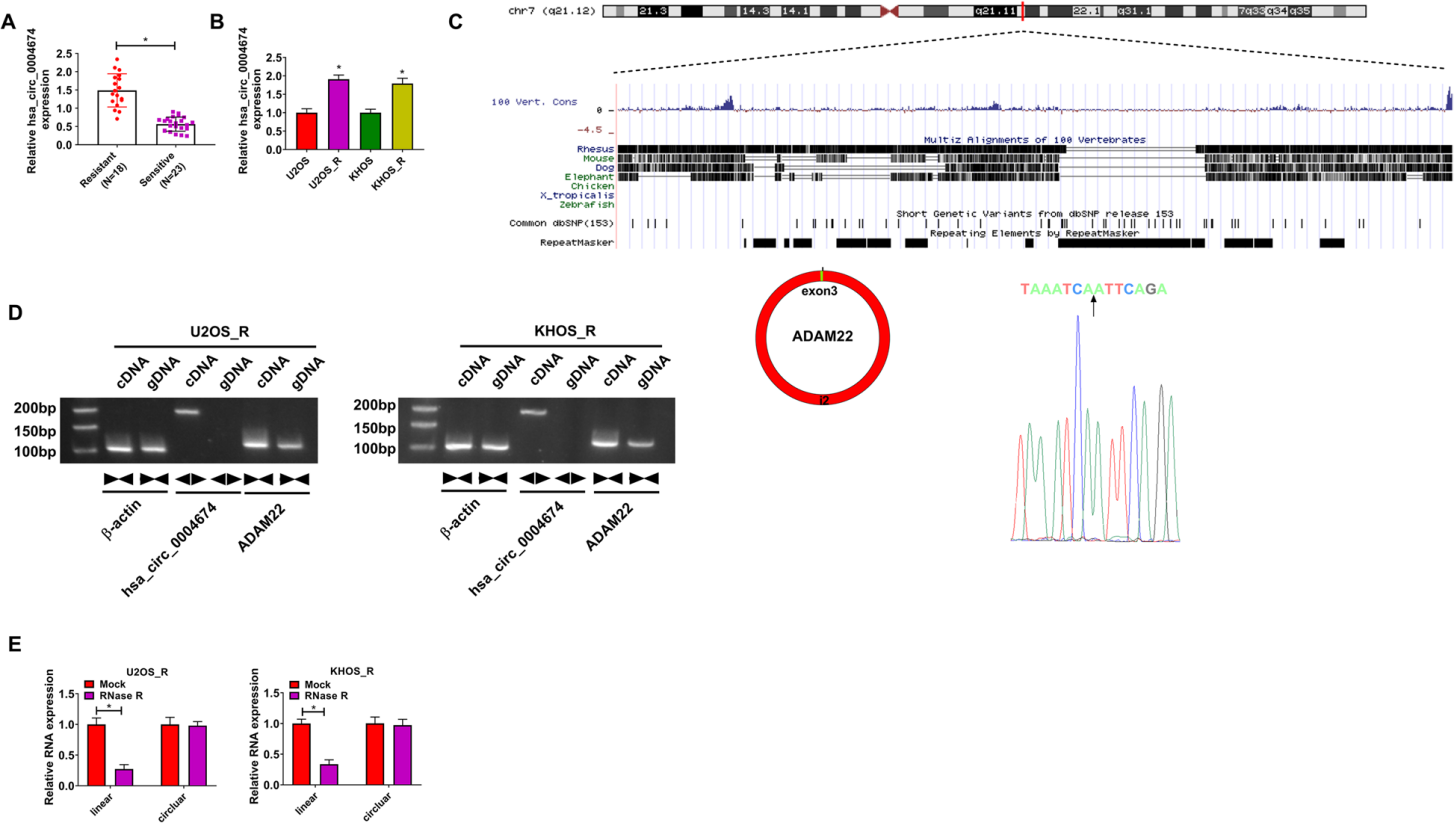
The hsa_circ_0004674 expression in DXR-resistant OS tissues and cells. **A** Hsa_circ_0004674 expression in DXR-resistant and DXR-sensitive OS tissues was measured by qRT-PCR. **B** QRT-PCR was performed to detect hsa_circ_0004674 expression in DXR-resistant OS cells (U2OS_R and KHOS_R) and normal OS cells (U2OS and KHOS). **C** The formation diagram of hsa_circ_0004674 was shown, and its splicing site was verified by Sanger sequencing. **D** After amplified by divergent primers and convergent primers in cDNA and gDNA using qRT-PCR, the PCR productions were detected by agarose gel electrophoresis. **E** The stability of hsa_circ_0004674 was examined by RNase R assay. *P < 0.05

In DXR-resistant and DXR-sensitive OS tissues, we discovered that hsa_circ_0004674 expression was remarkably higher in DXR-resistant OS tissues (Fig. 1A). The correlation analysis between has_circ_0004674 expression and clinicopathological characteristics of OS patients showed that high expression of has_circ_0004674 was associated with Enneking stage and chemoresistance in OS patients (Table 1). In addition, hsa_circ_0004674 was upregulated in DXR-resistant OS cells (U2OS_R and KHOS_R) compared to U2OS and KHOS cells (Fig. 1B). Through analysis, we confirmed that hsa_circ_0004674 is formed by the back-splicing of ADAM22 gene, as confirmed by Sanger sequencing (Fig. 1C). After amplified by convergent primers and divergent primers, we found that hsa_circ_0004674 could be amplified by divergent primers in cDNA but not in gDNA, while linear RNA β-actin and ADAM22 could be amplified by convergent primers in both cDNA and gDNA (Fig. 1D), which suggested that hsa_circ_0004674 had circular structure. Using the RNase R assay, we confirmed that hsa_circ_0004674 could resist the digestion of RNase R compared to its linear mRNA ADAM22 (Fig. 1E).

### Hsa_circ_0004674 knockdown reduced OS DXR resistance

**Fig. 2:**
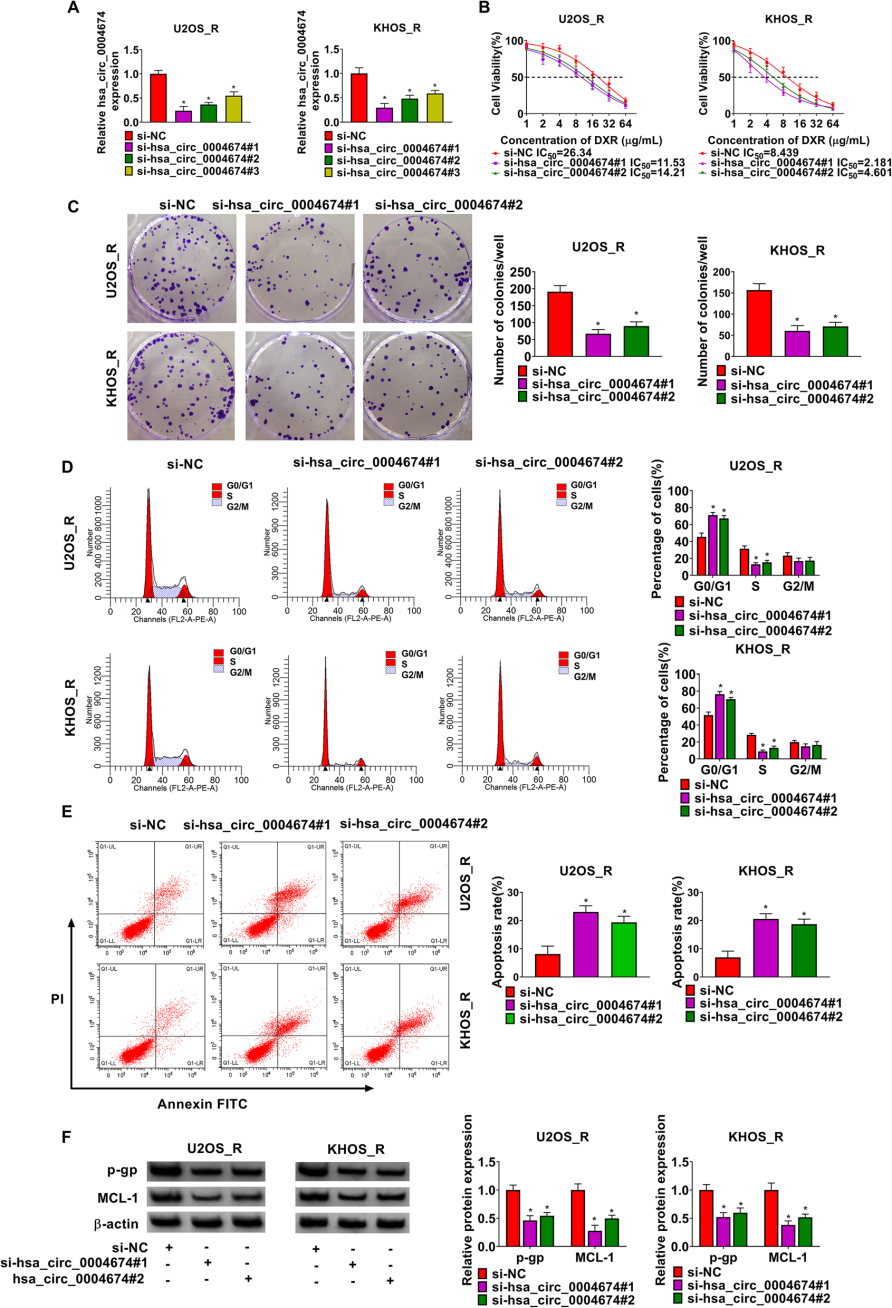
Hsa_circ_0004674 knockdown reduced OS DXR resistance. **A** The transfection efficiencies of three siRNAs for hsa_circ_0004674 were evaluated by detecting hsa_circ_0004674 expression using qRT-PCR in U2OS_R and KHOS_R cells. **B-F** U2OS_R and KHOS_R cells were transfected with si-NC, si-hsa_circ_0004674#1 or si-hsa_circ_0004674#2. **B** CCK8 assay was used to test the cell DXR resistance. Colony formation assay (**C**) and flow cytometry (**D-E**) were performed to determine the number of colonies, cell cycle process and apoptosis rate. **F** The p-gp and MCL-1 protein levels were tested by WB analysis. *P < 0.05

To identify the function of hsa_circ_0004674 in OS chemoresistance, three siRNAs for hsa_circ_0004674 were transfected into U2OS_R and KHOS_R cells to silence hsa_circ_0004674 expression. The results suggested that all three siRNAs could significantly reduce the expression of hsa_circ_0004674, especially si-hsa_circ_0004674#1 and si-hsa_circ_0004674#2 had better effects (Fig. 2A), so both were chosen for functional research. The IC_50_ of U2OS_R and KHOS_R cells after hsa_circ_0004674 knockdown was obviously decreased (Fig. 2B). Moreover, hsa_circ_0004674 silencing inhibited the number of colonies in OS cells (Fig. 2C), and promoted the cell number in G0/G1 phase to inhibit the cell number in S phase (Fig. 2D). These data indicated that OS cell proliferation was suppressed by hsa_circ_0004674 downregulation. In addition, hsa_circ_0004674 knockdown also promoted the apoptosis rate of U2OS_R and KHOS_R cells (Fig. 2E), and inhibited resistance markers p-gp and MCL-1 protein levels (Fig. 2F).

### Hsa_circ_0004674 sponged miR-342-3p

**Fig. 3:**
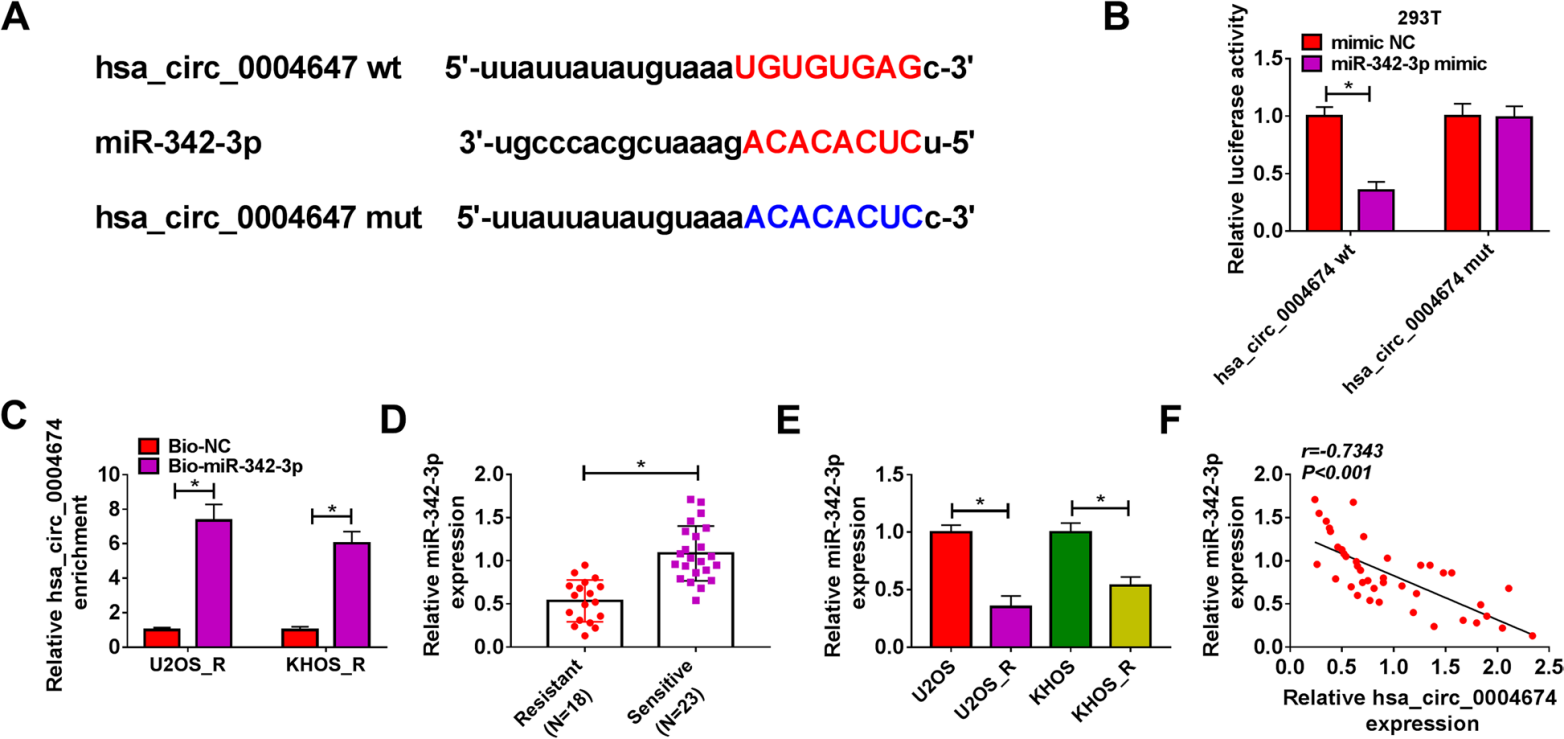
Hsa_circ_0004674 sponged miR-342-3p. **A** The target relationship between hsa_circ_0004674 and miR-342-3p was predicted by Starbase software. Dual-luciferase reporter assay (**B**) and RNA pull-down assay (**C**) were performed to assess the interaction between hsa_circ_0004674 and miR-342-3p. **D** QRT-PCR was used to examine the expression of miR-342-3p in DXR-resistant OS tissues and DXR-sensitive OS tissues. **E** MiR-342-3p expression in DXR-resistant OS cells (U2OS_R and KHOS_R) and normal OS cells (U2OS and KHOS) was measured by qRT-PCR. **F** Pearson’s correlation coefficient was employed to assess the correlation between hsa_circ_0004674 and miR-342-3p in OS tissues. *P < 0.05

In order to explore the potential mechanism of hsa_circ_0004674, we conducted bioinformatics analysis. The analysis result of starBase software showed that miR-342-3p had binding sites for hsa_circ_0004674. Then, we constructed the hsa_circ_0004674 wt and hsa_circ_0004674 mut vectors (Fig. 3A). MiR-342-3p mimic could specifically reduce the luciferase activity of hsa_circ_0004674 wt vector without affecting that of the mut vector (Fig. 3B). In addition, RNA pull-down assay results concluded that hsa_circ_0004674 could be significantly enriched in the Bio-miR-342-3p probe compared to that in Bio-NC probe (Fig. 3C). These data confirmed the interaction between hsa_circ_0004674 and miR-342-3p. Furthermore, miR-342-3p was found to be downregulated in DXR-resistant OS tissues and cells (Fig. 3D-E), and was also negatively correlated with hsa_circ_0004674 expression in OS tissues (Fig. 3F).

### MiR-342-3p inhibitor abolished the regulation of hsa_circ_0004674 silencing on OS DXR resistance

**Fig. 4:**
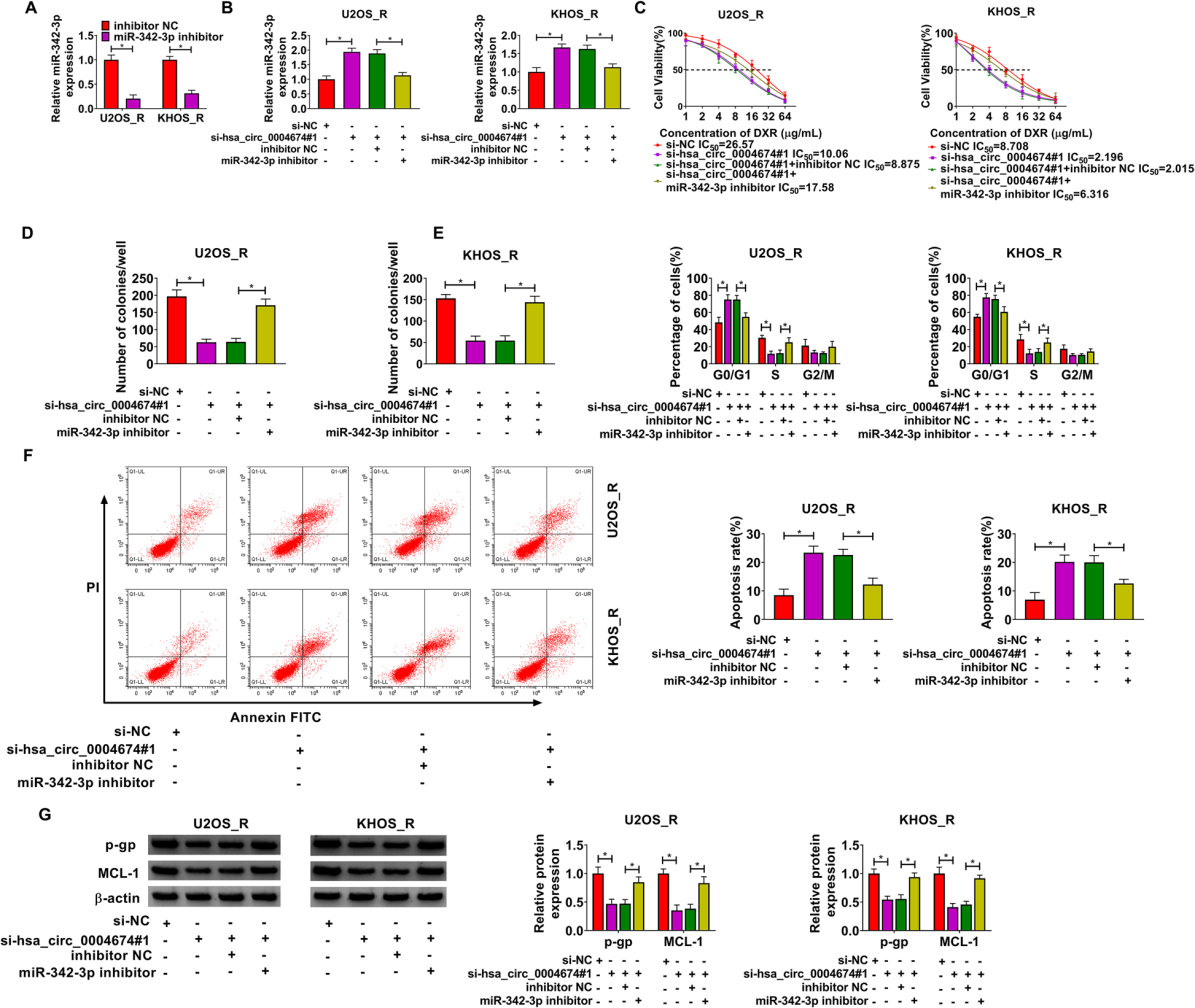
Hsa_circ_0004674 regulated OS DXR resistance by sponging miR-342-3p. **A** The transfection efficiency of miR-342-3p inhibitor was confirmed by detecting miR-342-3p expression in U2OS_R and KHOS_R cells using qRT-PCR. **B** MiR-342-3p expression was detected by qRT-PCR. **C** The DXR resistance of cells was assessed by CCK8 assay. The number of colonies, cell cycle process and apoptosis rate of cells were determined using colony formation assay (**D**) and flow cytometry (**E–F**). **G** WB analysis was performed to examine p-gp and MCL-1 protein levels. *P < 0.05

Subsequently, we explored whether hsa_circ_0004674 regulated OS chemoresistance by miR-342-3p. The significant decrease in miR-342-3p expression confirmed the transfection effectiveness of miR-342-3p inhibitor (Fig. 4A). Then, si-hsa_circ_0004674#1 and miR-342-3p inhibitor were co-transfected into OS cells. The promotion effect of si-hsa_circ_0004674#1 on miR-342-3p expression could be effectively restored by miR-342-3p inhibitor (Fig. 4B). CCK8 assay suggested that the IC_50_ reduced by hsa_circ_0004674 silencing could be recovered by miR-342-3p inhibitor (Fig. 4C). The suppressive effects of hsa_circ_0004674 knockdown on the number of colonies and cell cycle process in U2OS_R and KHOS_R cells also were overturned by miR-342-3p inhibitor (Fig. 4D-E). Additionally, miR-342-3p inhibitor also abolished the promotion effect of hsa_circ_0004674 knockdown on cell apoptosis rate (Fig. 4F), as well as the inhibitory effect on the protein levels of p-gp and MCL-1 in U2OS_R and KHOS_R cells (Fig. 4G). All results revealed that hsa_circ_0004674 sponged miR-342-3p to regulate OS DXR resistance.

### FBN1 was targeted by miR-342-3p

**Fig. 5:**
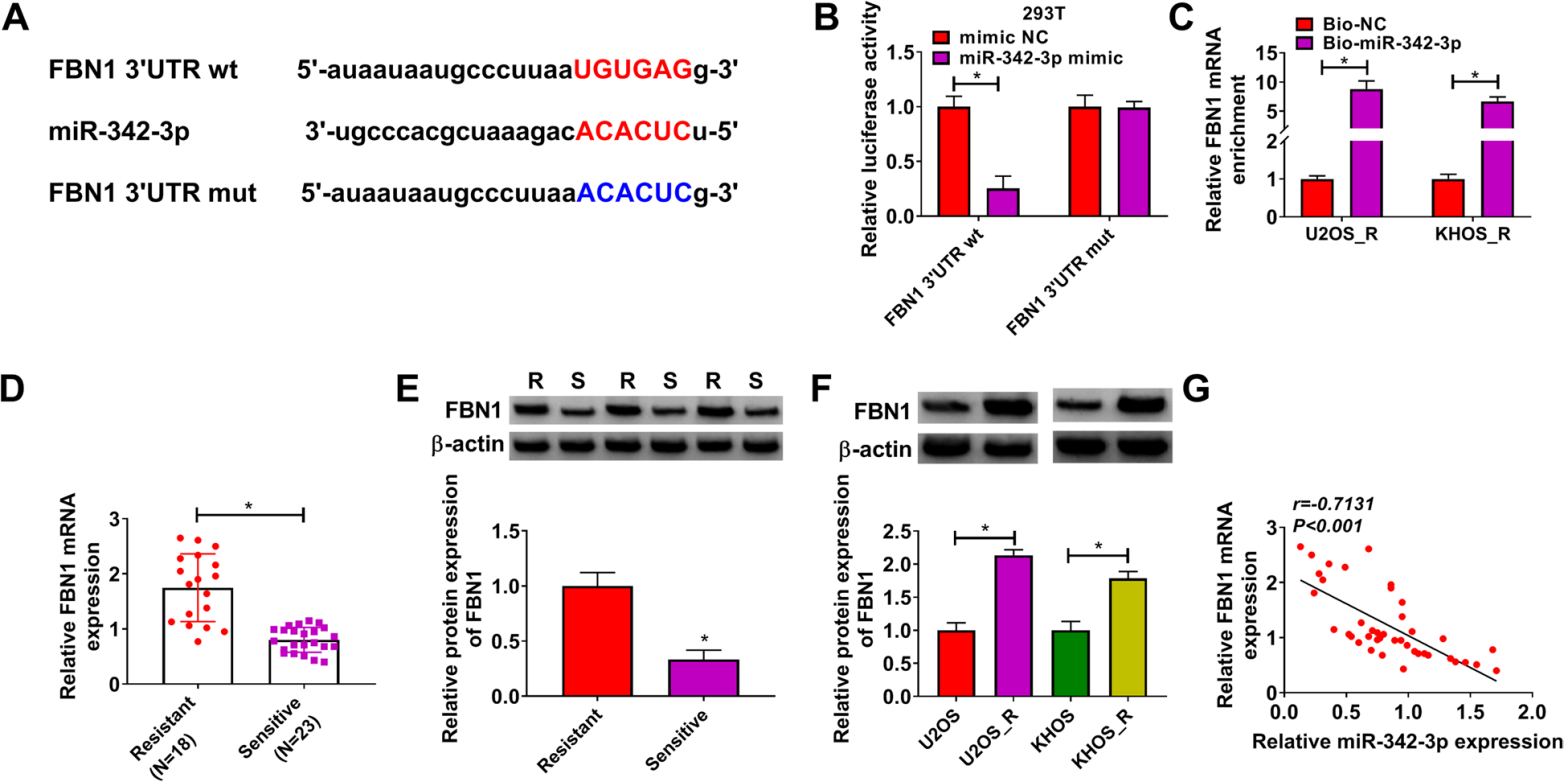
FBN1 was targeted by miR-342-3p. **A** The binding sites between miR-342-3p and FBN1 3’UTR was predicted by Starbase software. The interaction between miR-342-3p and FBN1 was detected by dual-luciferase reporter assay (**B**) and RNA pull-down assay (**C**). **D-E** FBN1 mRNA and protein expression levels in DXR-resistant OS tissues and DXR-sensitive OS tissues were measured by qRT-PCR and WB analysis. **F** The FBN1 protein expression in DXR-resistant OS cells (U2OS_R and KHOS_R) and normal OS cells (U2OS and KHOS) was detected by WB analysis. **G** The correlation between miR-342-3p and FBN1 in OS tissues was analyzed by Pearson’s correlation coefficient. *P < 0.05

Similarly, we also used the Starbase software to predict the targets of miR-342-3p and the 3’UTR of FBN1 was discovered to have binding sites for miR-342-3p (Fig. 5A). MiR-342-3p overexpression could inhibit the luciferase activity of FBN1 3’UTR wt vector, while had not effect on that of the mut vector (Fig. 5B). And the enrichment of FBN1 in the Bio-miR-342-3p probe was higher than the control probe (Fig. 5C). Further, we found a significantly high expression of FBN1 in DXR-resistant OS tissues at the mRNA and protein levels (Fig. 5D-E). Compared to normal OS cells (U2OS and KHOS), FBN1 protein expression in DXR-resistant OS cells (U2OS_R and KHOS_R) was notably increased (Fig. 5F). Additionally, FBN1 expression was negatively correlated with miR-342-3p expression in OS tissues (Fig. 5G).

### FBN1 reversed the regulation of miR-342-3p on OS DXR resistance

**Fig. 6:**
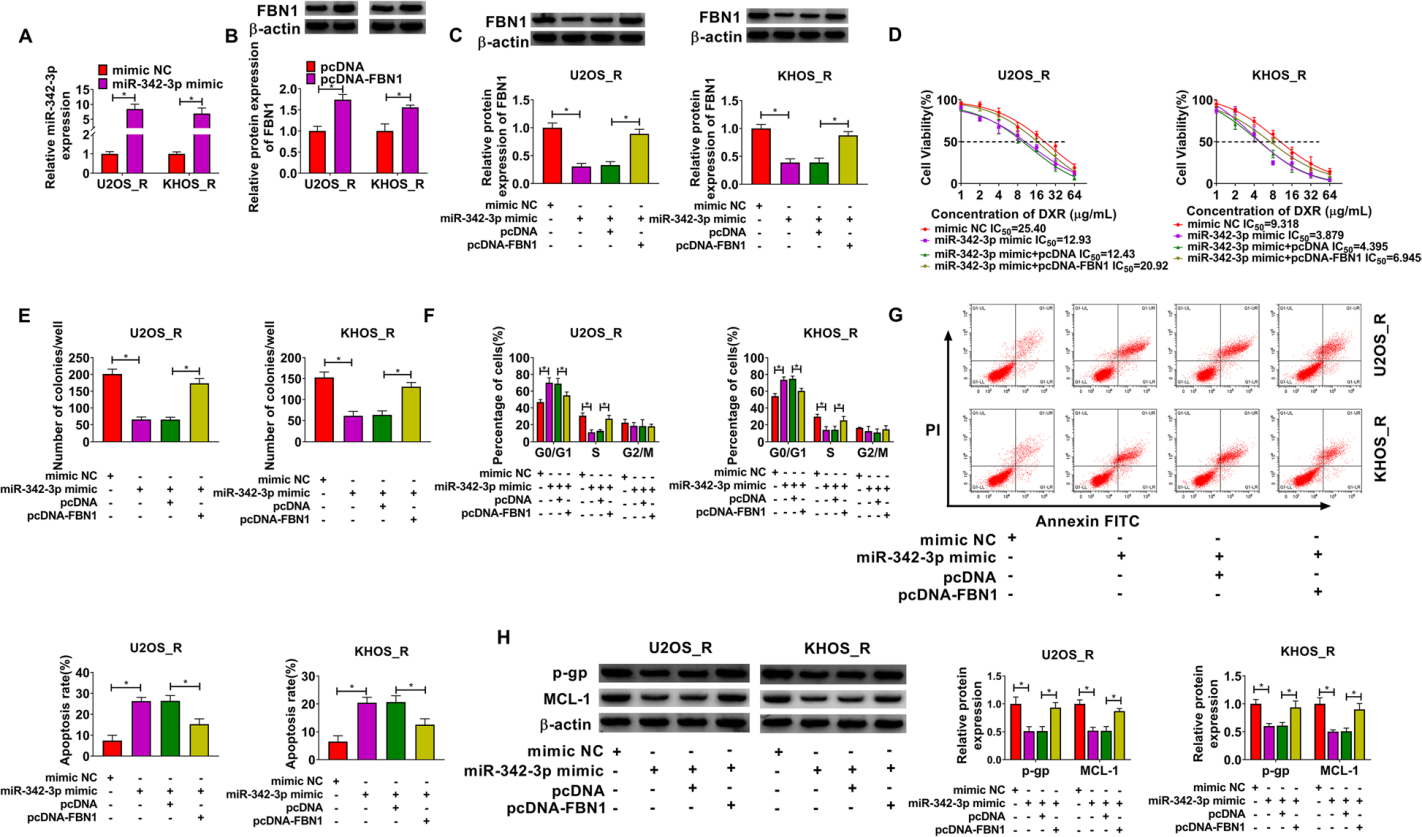
MiR-342-3p regulated OS DXR resistance by targeting FBN1. **A-B** MiR-342-3p and FBN1 expression was measured using qRT-PCR and WB analysis, respectively. **C** WB analysis was used to test FBN1 protein. **D** CCK8 assay was performed to detect the DXR resistance of cells. Colony formation assay (**E**) and flow cytometry (**F-G**) were used to analyze the number of colonies, cell cycle process and apoptosis rate. **H** The p-gp and MCL-1 protein levels were examined using WB analysis. *P < 0.05

To further investigate whether FBN1 participated in the regulation of miR-342-3p on OS DXR resistance, we performed rescue experiments. MiR-342-3p mimic could significantly promote miR-342-3p expression (Fig. 6A), and pcDNA-FBN1 could remarkably enhance the protein expression of FBN1 (Fig. 6B). After co-transfecting, we found that the decreasing effect of miR-342-3p mimic on FBN1 protein expression could be abolished by overexpressing FBN1 (Fig. 6C). MiR-342-3p suppressed the IC_50_, and this effect could be recovered by the addition of pcDNA-FBN1 (Fig. 6D). Meanwhile, miR-342-3p mimic hindered the number of colonies and induced cell cycle arrest, while FBN1 overexpression also could overturn these effects (Fig. 6E-F). Furthermore, overexpressed FBN1 also abolished the promotion effect of miR-342-3p on apoptosis rate and the inhibitory effect on the protein levels of p-gp and MCL-1 in U2OS_R and KHOS_R cells (Fig. 6G-H). Our results illuminated that miR-342-3p restrained the DXR resistance of OS cells by targeting FBN1.

### The hsa_circ_0004674/miR-342-3p/FBN1 axis regulated the activity of Wnt/β-catenin pathway

**Fig. 7:**
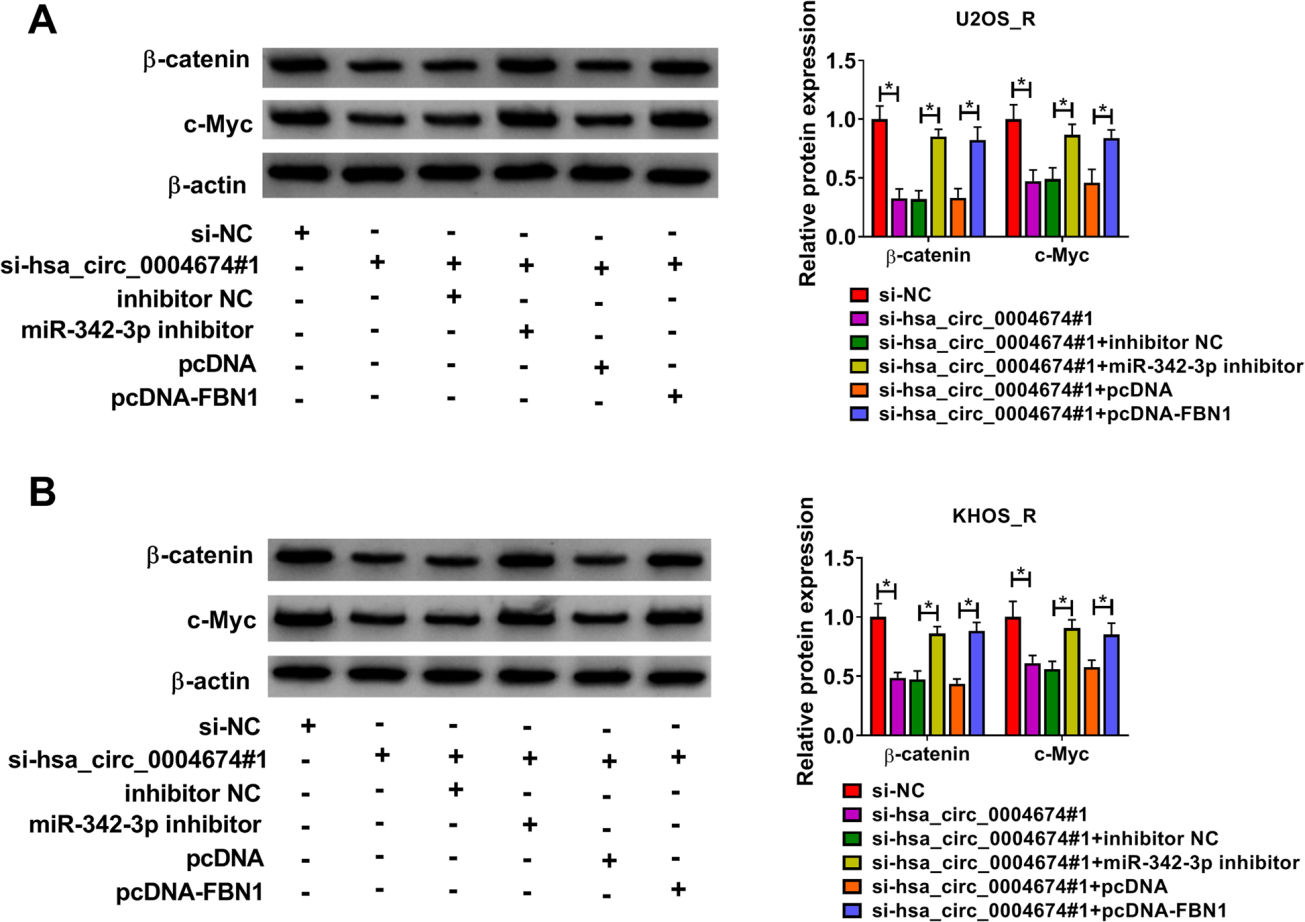
The hsa_circ_0004674/miR-342-3p/FBN1 axis regulated Wnt/β-catenin pathway activity. **A-B** The β-catenin and c-Myc protein expression levels were detected by WB analysis in U2OS_R and KHOS_R cells. *P < 0.05

Wnt/β-catenin pathway is involved in cancer cell chemoresistance [16, 17]. To evaluate whether the hsa_circ_0004674/miR-342-3p/FBN1 axis also regulated the activity of the Wnt/β-catenin pathway, we detected β-catenin and c-Myc protein expression. As shown in Fig. 7A-B, WB analysis revealed that hsa_circ_0004674 silencing could obviously inhibit β-catenin and c-Myc protein levels. However, the addition of miR-342-3p inhibitor or pcDNA-FBN1 reversed the suppressive effect of silenced-hsa_circ_0004674 on β-catenin and c-Myc protein levels. These data revealed that hsa_circ_0004674 could enhance Wnt/β-catenin pathway activity via regulating the miR-342-3p/FBN1 axis.

### Interference of hsa_circ_0004674 enhanced DXR sensitive of OS tumors

**Fig. 8:**
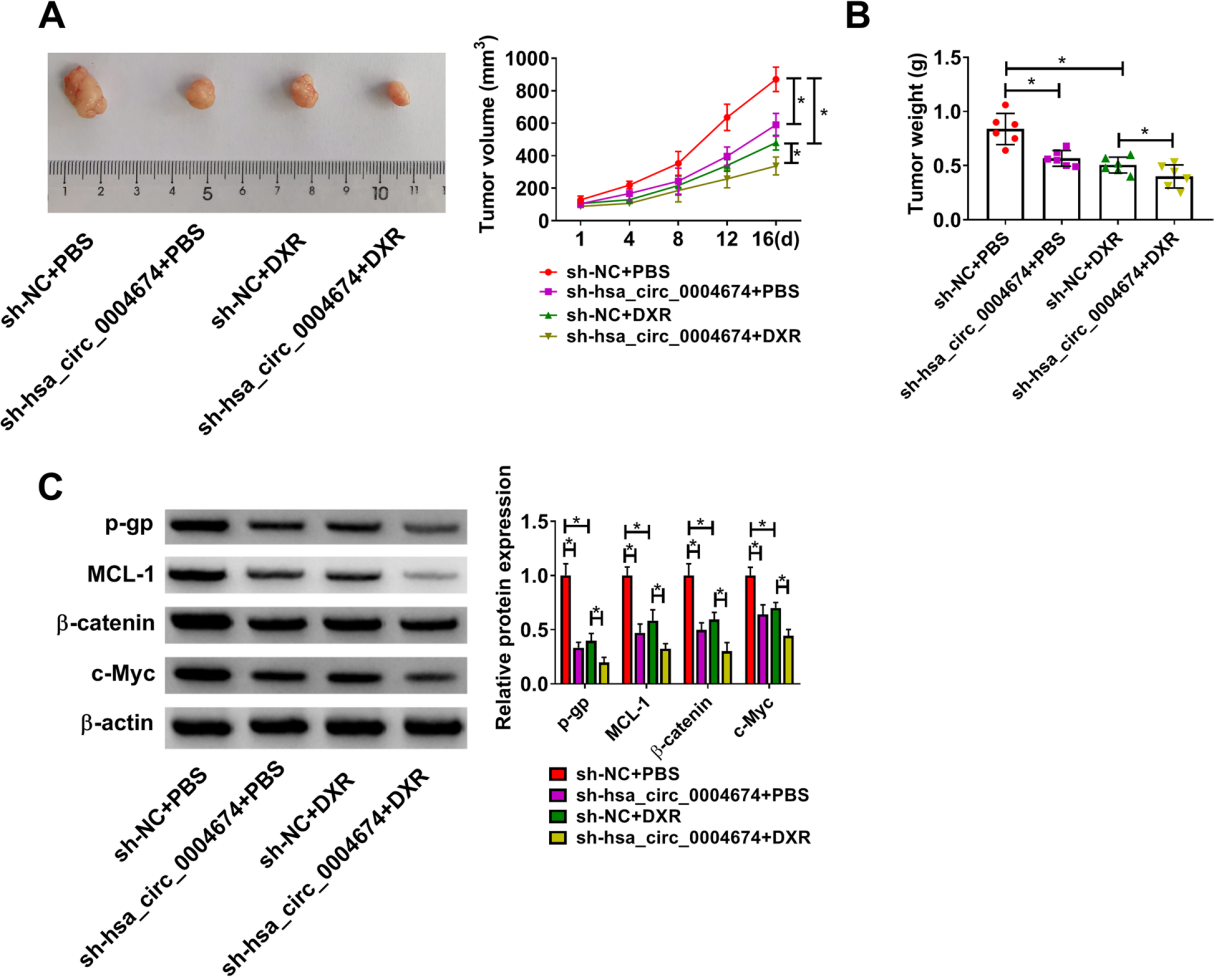
Interference of hsa_circ_0004674 enhanced DXR sensitive of OS tumors. Tumor volume (**A**) and tumor weight (**B**) in each group were determined. **C** The p-gp, MCL-1, β-catenin and c-Myc protein levels were measured by WB analysis. *P < 0.05

In addition, we conducted animal experiments to assess the role of hsa_circ_0004674 in the DXR resistance of OS tumors. Through measuring the tumor weight and tumor volume of OS, we found that hsa_circ_0004674 silencing or DXR treatment could markedly reduce the tumor volume and weight (Fig. 8A-B). In the DXR treatment group, hsa_circ_0004674 knockdown had a more obvious inhibitory effect on tumor growth, suggesting that hsa_circ_0004674 silencing could promote the DXR sensitive of OS tumors (Fig. 8A-B). Additionally, the protein expression levels of resistance markers (p-gp and MCL-1) and Wnt/β-catenin pathway markers (β-catenin and c-Myc) in OS tumor tissues were significantly decreased by hsa_circ_0004674 knockdown and DXR treatment (Fig. 8C).

## Discussion

DXR is an anti-tumor antibiotic, which is often used as the treatment of most malignant tumors in clinic, including OS, liver cancer, gastric cancer, and breast cancer [18, 19]. At present, many circRNAs have been found to participate in regulating OS chemoresistance. For example, hsa_circ_0000073 could promote the methotrexate resistance of OS [20], and circPVT1 regulated the DXR and cisplatin resistance of OS [21]. Hsa_circ_0004674 is a newly discovered circRNA. Consistent with the results of previous analyses [14], we discovered a high hsa_circ_0004674 expression in OS DXR-resistant cells and tissues. Hsa_circ_0004674 knockdown could lead to the decreased DXR resistance, reduced proliferation and increased apoptosis of OS cells. Silencing of hsa_circ_0004674 enhanced the sensitivity of OS tumors to DXR. These data indicated that the increased hsa_circ_0004674 expression was essential for maintaining the DXR resistance of OS, suggesting that hsa_circ_0004674 knockdown might be an effective way to treat OS chemoresistance.

MiR-342-3p is considered to be a suppressor in many tumors, including cervical cancer [22], hepatocellular carcinoma [23] and glioma [24]. Importantly, low miR-342-3p expression was related to tumor chemoresistance. MiR-342-3p could repress colorectal cancer chemoresistance [25], and could act synergistically with miR-205-5p to reduce tumor chemoresistance by inhibiting E2F1 [26]. MiR-342-3p had an obvious tendency of low expression in OS, and has been proved to inhibit the progression and promote the radiosensitivity of OS [27–29]. Our study discovered that miR-342-3p was underexpressed in the DXR-resistant OS tissues and cells, and it also hindered OS DXR resistance. The reversal effect of anti-miR-342-3p on si-hsa_circ_0004674 suggested that hsa_circ_0004674 regulated the DXR resistance of OS via targeting miR-342-3p.

Further experiments revealed that miR-342-3p targeted FBN1. FBN1 is the main component of extracellular matrix microfibrils, which can maintain the integrity and normal function of connective tissue [30, 31]. In addition to Marfan syndrome caused by FBN1 mutations, many studies have also reported that abnormal FBN1 expression is related to tumor malignant phenotype, including ovarian cancer and papillary thyroid carcinoma [32, 33]. Liu et al*.* showed that FNB1 was upregulated in OS, and its knockdown could inhibit OS tumorigenesis [34]. Therefore, FNB1 also had a positive role in OS progression. Our data also revealed that miR-342-3p promoted the DXR resistance by targeting FBN1.

Wnt/β-catenin is one of the important signaling pathways that mediate embryonic development and cell biological functions, and it is abnormally activated in a variety of human diseases [35]. Studies have shown that the activity of the Wnt/β-catenin pathway is significantly up-regulated in the malignant progression of many cancers [36]. Targeted inhibition of Wnt/β-catenin pathway is considered to be an effective way of cancer treatment, including OS [37, 38]. In the related studies of OS, activation of Wnt/β-catenin pathway has been found to be related to chemoresistance of OS, and inhibition of Wnt/β-catenin pathway has been proved to improve the chemotherapy sensitivity of OS [39, 40]. In this, we discovered that silencing of hsa_circ_0004674 could inhibit the activity of the Wnt/β-catenin pathway. Further analysis showed that hsa_circ_0004674 positively regulated Wnt/β-catenin pathway activity through the miR-342-3p/FBN1 axis. These results are a new discovery for us.

## Conclusion

In conclusion, our experiment confirmed the function of hsa_circ_0004674 in OS chemoresistance for the first time. We found that hsa_circ_0004674 could facilitate OS chemoresistance via miR-342-3p/FBN1 axis to activate the Wnt/β-catenin pathway. Our research provided a theoretical basis for overcoming the chemoresistance of OS.

## Data Availability

Not applicable.

## Abbreviations

circRNA: Circular RNA
OS: Osteosarcoma
FBN1: Fibrillin-1
CCK8: Cell counting kit 8
DXR: Doxorubicin
WB: Western blot
